# Reduction of Beany Flavor and Improvement of Nutritional Quality in Fermented Pea Milk: Based on Novel *Bifidobacterium animalis* subsp. *lactis* 80

**DOI:** 10.3390/foods13132099

**Published:** 2024-07-01

**Authors:** Ronghao Sun, Bochun Yang, Conghao Yang, Yan Jin, Wenjie Sui, Guohua Zhang, Tao Wu

**Affiliations:** 1 Engineering Research Center of Food Biotechnology, Ministry of Education, College of Food Science and Engineering, Tianjin University of Science & Technology, Tianjin 300457, China; sunronghao1998@163.com (R.S.); bcyang0810@126.com (B.Y.); yangconghao@mail.tust.edu.cn (C.Y.); jinyan@tust.edu.cn (Y.J.); sjsui@tust.edu.cn (W.S.); 2 School of Life Sciences, Shanxi University, Taiyuan 030006, China; zhanggh@sxu.edu.cn

**Keywords:** pea milk, lactic acid bacteria, fermentation, GC-MS, volatile compounds

## Abstract

Peas (*Pisum sativum* L.) serve as a significant source of plant-based protein, garnering consumer attention due to their high nutritional value and non-GMO modified nature; however, the beany flavor limits its applicability. In this study, the effects of *Bifidobacterium* animalis *subsp. Lactis* 80 (Bla80) fermentation on the physicochemical characteristics, particle size distribution, rheological properties, and volatile flavor compounds of pea milk was investigated. After fermentation by Bla80, the pH of pea milk decreased from 6.64 ± 0.01 to 5.14 ± 0.01, and the (D_4,3_) distribution decreased from 142.4 ± 0.47 μm to 122.7 ± 0.55 μm. In addition, Lactic acid bacteria (LAB) fermentation significantly reduced the particle size distribution of pea milk, which was conducive to improving the taste of pea milk and also indicated that Bla80 had the probiotic potential of utilizing pea milk as a fermentation substrate. According to GC-MS analysis, 64 volatile compounds were identified in fermented pea milk and included aldehydes, alcohols, esters, ketones, acids, and furans. Specifically, aldehydes in treated samples decreased by 27.36% compared to untreated samples, while esters, ketones, and alcohols increased by 11.07%, 10.96%, and 5.19%, respectively. These results demonstrated that Bla80 fermentation can significantly decrease the unpleasant beany flavor, such as aldehydes and furans, and increase fruity or floral aromas in treated pea milk. Therefore, Bla80 fermentation provides a new method to improve physicochemical properties and consumer acceptance of fermented pea milk, eliminating undesirable aromas for the application of pea lactic acid bacteria beverage.

## 1. Introduction

Peas (*Pisum sativum* L.) are one of the most cultivated pulses in the world, with good adaptability and wide geographical distribution, being grown in more than 85 countries [1]. According to the Food and Agriculture Organisation of the United Nations (FAO), the global pea harvest area in 2022 is 9,749,400 hectares with a total production of 34,535,900 tons. Peas are rich in protein, carbohydrates, insoluble dietary fiber, and minerals and are relatively low in fat [2]. Pea protein is rich in amino acids and has a balanced composition [3], which is favored by consumers and food developers because of its low allergic reaction [4] and high nutritional value [2]. Compared with cereal protein, pea protein has a relatively high content of lysine, phenylalanine, and leucine and low levels of sulfur-containing amino acids. Therefore, pea resource development is considered beneficial to health and has good development prospects. The demand for healthy and low-carbon food products is gradually increasing, with plant-based beverages being preferred over animal-based alternatives due to their lower carbon footprint. Peas, favored by consumers for being lactose-free and nutritionally rich, hold the potential for the development of pea-based plant protein beverages. However, a key technological challenge in current development efforts lies in addressing the distinct beany flavor inherent to peas [5].

The accumulation of aromas and flavors imbues the product with its distinctive taste profile. Previous studies have often characterized the beany flavor of peas as green, unpleasant, earthy, and fatty [6]. Key flavor compounds in peas, such as hexanal, 1-hexanol, 2-pentylfuran, and 1-heptanal, contribute collectively to their unpleasant beany taste [7]. This undesirable flavor attribute primarily stems from the degradation of fatty acids present in peas [8]. Unsaturated fatty acids, including linoleic acid and linolenic acid, undergo various pathways, such as autoxidation, photooxidation, and enzymatic oxidation, resulting in the generation of volatile compounds. This process involves lipoxygenases (LOX) catalyzing the conversion of polyunsaturated fatty acids into hydroperoxides, which can further decompose to yield the volatile substances responsible for the characteristic odor in peas [9].

Compared with soybeans, peas have hypoallergenic and non-GMO characteristics, but the unpleasant bean taste seriously limits its application in food and also limits the development of innovative foods. LAB, a type of microorganism frequently utilized in fermented food production, are recognized for their research efficacy and safety, garnering considerable attention for their ability to enhance food aroma and improve desirable sensory attributes [10]. Fermentation serves as a pivotal processing technique that not only mitigates unpleasant flavors but also imparts unique fermented notes during bean processing [11]. This transformative process involves the breakdown and formation of organic compounds, leading to the development of intensified flavors [12]. Simultaneously, lactic acid fermentation can reduce the content of oligosaccharides in peas, alleviating the discomfort caused by intestinal microbial utilization of oligopeptides in the human body [13]. Presently, strains commonly employed for the fermentation of beans encompass a variety of bacterial species, including *Lactobacillus rhamnosus*, *Lactobacillus plantarum*, *Lactobacillus acidophilus*, and *Lactobacillus casei*. Li et al. [14] found that fermentation of yellow pea flour using *Lactobacillus acidophilus* could produce new aromatic compounds that contributed to the improvement of the flavor profile. Schindler et al. [15] demonstrated that *L. plantarum* fermentation has the potential to ameliorate the unpleasant beany flavor profile of pea protein. Zhang et al. [16] showed that by using *Lactobacillus rhamnosus* to ferment chickpeas, the new flavor compounds produced during fermentation can reduce and/or mask the original odor. In summary, LAB fermentation can improve the sensory flavor and nutritional quality of food [17]. Hence, the advancement of fermentation processes and fermented products holds significance in enhancing the nutritional value and consumer acceptance of plant-based dairy alternatives [18]. *Bifidobacterium animalis* subsp. *lactis* is a commonly occurring microorganism that colonizes the mammalian gut and has extensive application in food production, particularly in fermented dairy products. Extensive animal and clinical studies have highlighted the ability of *Bifidobacterium animalis* subsp. *lactis* to modulate gut microbiota equilibrium, boost host immune function, aid in nutrient metabolism and absorption, and enhance gastrointestinal functionality, thereby promoting overall gut health [19]. There is a paucity of relevant research pertaining to the utilization of *Bifidobacterium animalis* subsp. *lactis* fermentation for ameliorating the unpleasant flavor profile of peas.

In order to reduce unpleasant flavors, the present investigation aimed to examine the effect of Bla80 fermentation on physicochemical properties, rheological characteristics, and flavor substance of pea milk. This research aimed to evaluate the changes in volatile flavor compounds pre- and post-Bla80 fermentation of pea milk, with the objective of diminishing or eradicating the unpleasant taste associated with peas. Additionally, this research offers a theoretical foundation for the processing and research of Bla80 in plant-based milk.

## 2. Materials and Methods

### 2.1. Materials and Reagents

Peas were produced by Sichuan Hao Rui Gallium Biotechnology Co., Ltd. (Chengdu, China). *Bifidobacterium animalis subsp. lactis.* 80 (Bla80) was produced by Jiangsu Weikang Biotechnology Co., Ltd. (Suzhou, China). MRS Broth was kindly provided by Qingdao Hope Bio-Technology Co., Ltd. (Qingdao, China). 2-methyl-3-heptanone was bought from Shanghai Aladdin Biochemical Technology Co., Ltd. (Shanghai, China), and white granulated sugar was purchased from Angel Yeast Co., Ltd. (Yichang, China).

### 2.2. Preparation and Fermentation of Pea Milk

The preparation and fermentation of pea milk were based on the description of Zhang et al. [20] with some modifications. The peas were soaked in deionized water for 10 h. The skin of the soaked dry peas was gradually removed, and the shells were wet-dehulled under these conditions. The pea samples were grounded using a high-speed multifunctional crusher (SUPOR, SP36S, Shaoxing, China) with a ratio of 1:19 *w*/*w* deionized water for a duration of 24 min. The resulting mixture was then filtered through 80 mesh filters and is referred to as raw pea milk. Subsequently, the raw pea milk was subjected to boiling for 15 min, yielding unfermented pea milk (UFP). The UFP was further heated to 95 °C for 15 min and then cooled to approximately 38 °C. To initiate fermentation, the lyophilized strain powder (0.014% *w*/*w*) was mixed with the UFP for 30 s, after which the samples were fermented in a fermentation oven at 38 °C for a duration of 8 h, resulting in the formation of fermented pea milk (FP). Prior to freeze-drying, all samples underwent pH, titratable acidity, rheological properties, texture characteristics, and particle size distribution analysis. Freeze-dried samples were utilized for electronic nose analysis and flavor composition determination.

### 2.3. Fermentation Performance Determination

The pH value of fermented pea milk samples with Bla80 added was directly measured by a pH meter (pHS-3BW, Changzhou, China) every 1 h. The method for the determination of titratable acidity (% lactic acid) was based on the description of Li et al. [21]. Briefly, 10 mL of sample is transferred with 20 mL of deionized water into a 100 mL conical flask and carefully mixed. Subsequently, two droplets of phenolphthalein were introduced to the samples and titrated with 0.1 mol/mL NaOH until a consistent pink hue materialized.

### 2.4. Analysis of Texture Characteristics

Texture profile analysis (TPA) of pea milk before and after fermentation was conducted using a TA/BE probe (Stable Microsystems, London, UK) based on the method previously described, with minor modifications [22]. The samples were measured using a 40 mm diameter TA/BE cylinder probe analyzed for consistency, work of cohesion, hardness, and cohesiveness characterization. The diameter cylinder probe compresses the specimen to 70% deformation with a pretest speed of 4.0 mm/s, a test velocity of 2.0 mm/s, and a post-test speed of 2.0 mm/s. For each sample, three replications were performed.

### 2.5. Particle Size Distribution

The integrated laser light scattering instrument (Bettersize2600, Dandong, China) was used to measure the particle size distribution of the samples with reference to the method of Rui et al. [23]. The size of protein particles in pea milk was quantified by calculating the volume-weighted mean diameter (D_4,3_).

### 2.6. Determination of Rheological Properties

The rheological measurements of fermented pea milk were studied using a Haake Mars60 (Thermo Fisher Scientific, Dreieich, Germany) rheometer based on the method previously described, with minor modifications [24]. A 30 mm diameter stainless steel flat probe with a 1.0 mm gap was used to measure. A series of measurements were conducted on the samples at a controlled temperature of 25 ± 0.5 °C, as follows: (1) Oscillatory strain scanning experiments were performed over a range of strain amplitudes (0.1~10%) at a frequency of 1.0 Hz, with the objective of determining a consistent strain of 0.5%. (2) The variation of apparent viscosity with shear rate was analyzed within the range of 0.10~100.00 s^−1^. (3) Frequency sweep experiments were carried out from 0.1~100 Hz, with a fixed 0.5% strain. (4) The creep phase was recorded for 300 s under stress of 3 Pa, followed by a recovery phase recorded for 300 s at 0 Pa stress.

### 2.7. Electronic Nose Analysis

The flavor profile was conducted using the PEN 3 E-nose (Airsense Analytics, GmBH, Schwerin, Germany), and the sensors and applications used are listed in Table 1. The analytical method was carried out according to Yi et al. [11]. The methodology is as follows, with some modifications. A 5 g sample of either pea milk or fermented pea milk and 2 mL 20% (*w*/*v*) NaCl solution into a 20 mL headspace vial. The vial was then subjected to equilibration for a period of 30 min in a water bath heater set at 60 °C. The test is then performed using the E-nose. The samples were injected manually, with three replicates in parallel for each sample. The synthetic air flow rate was 150 mL/min, and the data acquisition time was 120 s with a 60 s acquisition delay.

### 2.8. Volatile Compounds Analysis

The method was carried out according to Gao et al. [25]. The methodology is as follows, with some modifications. Place 2.0 g of freeze-dried sample into 20 mL headspace vials. Subsequently, 5 mL of ultra-pure water, 10 μL of 2-methyl-3-heptanone, and 2 mL of 20% (*w*/*v*) NaCl solution were added. After the mixture is evenly mixed, it is balanced at 60 °C for 25 min on a magnetic stirrer equipped with heating and stirring functions. After equilibration, solid-phase microextraction (SPME) fiber (75 μm CAR/PDMS) was extended through the headspace vials, and headspace adsorption was performed at 60 °C for 45 min. The separation of the aroma compounds was implemented by utilizing a DBWAX capillary column (BRUKER, Billerica, MA, USA). The GC temperature program: initial temperature 40 °C; split ratio (R): 5.0; column flow rate: 2.00 mL/min. The MS was set as follows: inlet temperature: 220 °C; injector temperature: 250 °C; ion source temperature: 220 °C; mass scanning range: 40.0~500.0 m/z; and scanning speed: 1000. The detected peaks were compared with the standard mass spectra provided by NIST11s Library to identify the compounds. Only compounds with a matching degree greater than 85 were selected. The peak area was measured and normalized to the internal standard to determine the amount of each compound.

### 2.9. Statistical Analysis

One-way analysis of variance (ANOVA) was conducted using SPSS 24.0 software (Armonk, N.Y., USA). Origin 2019 program (Origin Lab Inc., Northampton, MA, USA) was used for data processing. A *p*-value < 0.05 was considered statistically significant. All experiments were performed three times. The data obtained were presented as mean values ± standard deviation (SD).

## 3. Results and Discussion

### 3.1. Fermentation Characteristics

**Figure 1:**
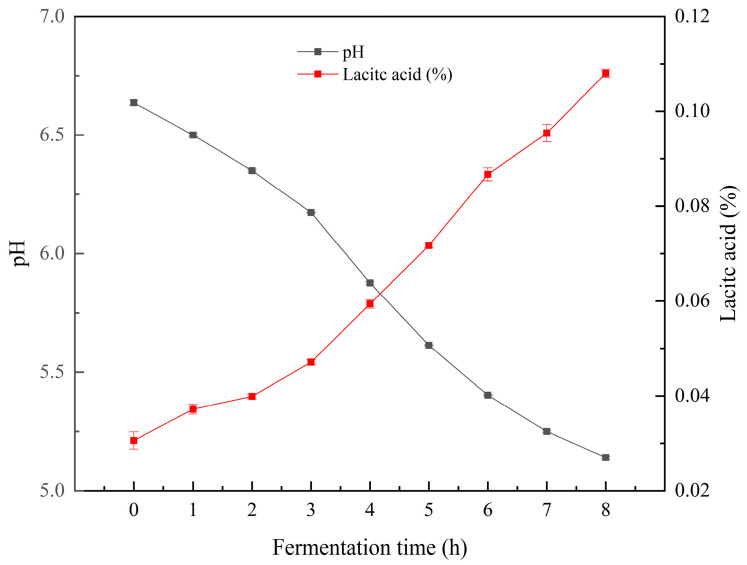
Fermentation characteristics: pH and titratable acidity (% lactic acid) of pea milk during fermentation.

The pH and titratable acidity (% lactic acid) of fermented pea milk cultured with Bla80 ferment are illustrated in Figure 1. Over the course of fermentation, there was a gradual decline in the pH of the pea milk. Specifically, the initial pH of 6.64 decreased to a final pH of 5.14 after 8 h of fermentation (Figure 1). At the conclusion of fermentation, the lactic acid content in pea milk was 0.11%. With prolonged fermentation, Bla80 fermentation generated a significant amount of lactic acid, with lactic acid being the quintessential byproduct of fermentation, ultimately leading to a decrease in pH value. The obtained results clearly showed that Bla80 could use pea milk as a substrate for growth. IRAPORDA et al. The authors of [26] examined the titratable acidity of soy-based formulations, represented as a percentage of lactic acid 8 h prior to fermentation at 37 °C, within the range of 0.07–0.15%, akin to the fermentation curve trend observed in this study. 

### 3.2. Analysis of Texture Characteristics

The texture analysis is mainly characterized by hardness, consistency, cohesiveness, and work of cohesion characterization. As can be seen from Table 2, the hardness of fermented pea milk was significantly higher than that of unfermented pea milk (*p <* 0.05). Previous research has indicated that the hardness reflects the gel strength of pea milk, suggesting that fermented pea milk exhibits greater gel strength compared to unfermented pea milk [27]. The exopolysaccharides (EPS) synthesized by probiotic bacteria can contribute to heightened viscosity, water retention, and other interactions within pea milk, ultimately leading to an increase in product hardness. The viscosity of fermented pea milk was notably higher than that of unfermented pea milk (*p* < 0.05), signifying that fermentation effectively enhanced the viscosity of pea milk. In terms of cohesiveness and work of cohesion index, there were no significant alterations observed before and after the fermentation of pea milk. RUI et al. [23] conducted an investigation on the fermentation of *L. plantarum* B1-6 to produce soy yogurt and found the same trend, which is similar to our findings.

### 3.3. Particle Size Distribution

**Figure 2:**
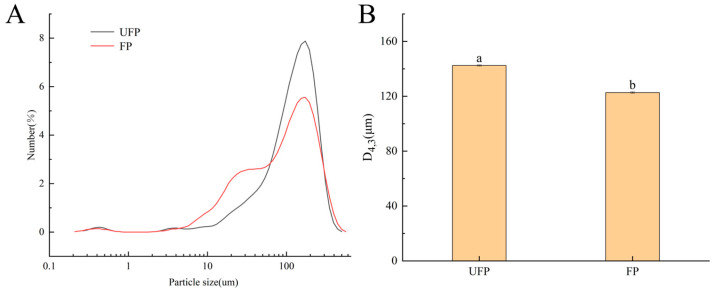
Changes of (**A**) particle size distribution and (**B**) volume mean particle size (D_4,3_) of pea milk during fermentation. UFP: unfermented pea milk; FP: fermented pea milk. Different superscript letters in the figure denote significant differences between samples (*p* < 0.05).

Figure 2A illustrates the alterations in particle size and distribution of pea milk before and after fermentation. The particle size of unfermented pea milk peaked at about 170 μm, while the particle number of fermented pea milk decreased significantly at about 170 μm. In the fermented pea milk, the number of particles in the range of 10~40 μm increased. Fermentation changes the particle size distribution of pea milk from a single peak to a dual peak distribution. 

The decrease in the grain size of soybean milk is beneficial to improving the taste of pea milk, the utilization rate of raw materials, and the digestibility of protein [28]. Figure 2B shows the (D_4,3_) distribution of pea milk before and after fermentation at 142.4 ± 0.47 μm and 122.7 ± 0.55 μm. The data showed that fermentation significantly decreased (D_4,3_) distribution of pea milk (*p* < 0.05). Fermentation can break down larger particles in pea milk into smaller ones, contributing to a more stable structural system in the beverage and enhancing its delicate texture. Liang et al. [29] showed that fermentation can change the particle size of mung soybean milk, with a significant reduction in (D_4,3_) after fermentation. 

### 3.4. Determination of Rheological Properties

**Figure 3:**
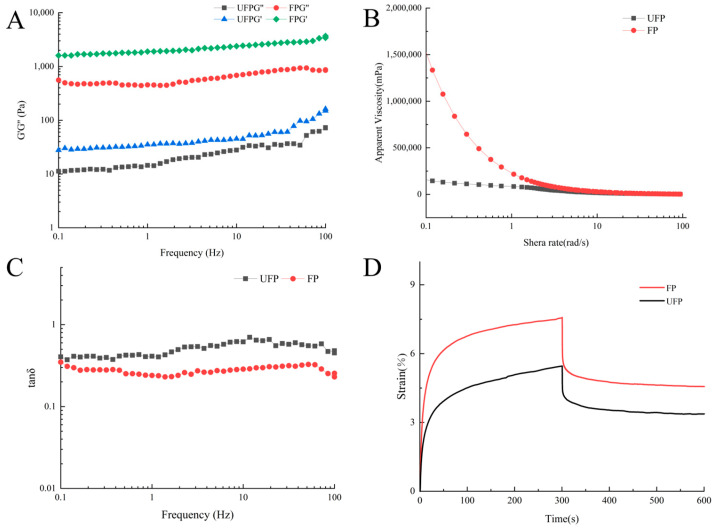
The rheological property analysis (G’, the storage modulus, and G”, the loss modulus) of fermented pea milk and pea milk. (**A**) Frequency sweep; (**B**) Relationship between apparent viscosity and shear rate; (**C**) tan δ; (**D**) Creep recovery curves. UFP: unfermented pea milk; FP: fermented pea milk.

Rheological properties play a crucial role in the processing and quality assurance of fermented milk beverages [30]. The frequency scan feature of a rheometer is commonly utilized to assess the viscoelastic behavior of the sample across varying scanning frequencies [31]. This method serves as an indirect simulation of human mastication. As depicted in Figure 3A, both the *G*′ (storage modulus) and *G*″ (loss modulus) of both pea milk and fermented pea milk displayed an increasing trend with rising frequency (0.1~100 Hz). Furthermore, *G*′ consistently exceeded *G*″ in both samples, indicating the prevalence of elastic components and suggesting solid characteristics post-ripening. These results imply that the viscoelastic properties of pea milk can be modified through the fermentation process [32]. The observed phenomenon may be attributed to the production of lactic acid during the fermentation process of the Bla80 strain, resulting in a gradual decrease in pH, diminishing the surface activity of pea protein molecules, fostering aggregation between protein particles, thereby elevating the viscosity of the fermented pea milk.

The correlation between apparent viscosity and shear rate is depicted in Figure 3B. Apparent viscosity is a critical parameter that significantly influences the taste and quality of Lactobacillus beverages. The results indicated an enhancement in the apparent viscosity of pea milk throughout the fermentation process. Figure 3B illustrates a decline in apparent viscosity for both pea milk and fermented pea milk as the shear rate increases. It was observed that within the same range of shear rates, the apparent viscosity of fermented pea milk exceeded that of unfermented pea milk. The viscosity of fermented pea milk exhibited a non-linear relationship with shear stress, suggesting its behavior as a non-Newtonian fluid [33].

The viscosity of fermented pea milk increases, which is mainly attributed to the enhanced availability of granular protein. In fact, the increase in apparent viscosity of the fermented pea milk is attributable to the accumulation of lactic acid as they ferment, which leads to a lower pH in the pea milk and thus promotes protein binding [30]. The interaction between exopolysaccharides (EPS) and proteins plays a crucial role in fermented milk products [32]. Studies have shown that *Bifidobacterium animalis* subsp. *Lactis* SF has the probiotic potential to produce exopolysaccharides [34]. Korma et al. [30] show that exopolysaccharides produced by LAB fermentation can improve the texture and organoleptic qualities of fermented milk.

Smaller values of the phase angle (tan δ) indicate a more robust solid structure throughout the sample, imparting a stronger network for gel formation. The tan δ value of fermented pea milk was lower than that of unfermented pea milk (Figure 3C), reflecting enhanced solid properties in the sample post-fermentation. The graph presented in Figure 3D illustrates the recovery curve of deformation in fermented pea milk before and after fermentation, depicting the variation in deformation quantity over time when the sample is subjected to a constant external force. The viscosity coefficient (η_1_) of fermented pea milk is higher than that of unfermented pea milk, indicating an increase in the viscosity of the pea milk and a more stable internal gel structure. These findings suggest that fermented pea milk demonstrates superior dynamic viscoelastic properties compared to unfermented pea milk following Bla80 fermentation.

During the fermentation process, the protein particles in the pea milk become smaller, and lactic acid is produced during the fermentation process, resulting in a gradual decrease in pH value. The surface activity of pea protein molecules decreases, and protein particles aggregate, contributing to the establishment of a more uniform and cohesive protein network structure, which increases the structural viscosity of pea milk. Smaller protein and fat particles, which are more evenly distributed throughout the drink, contribute to the stability of the beverage.

### 3.5. Electronic Nose Analysis

**Figure 4:**
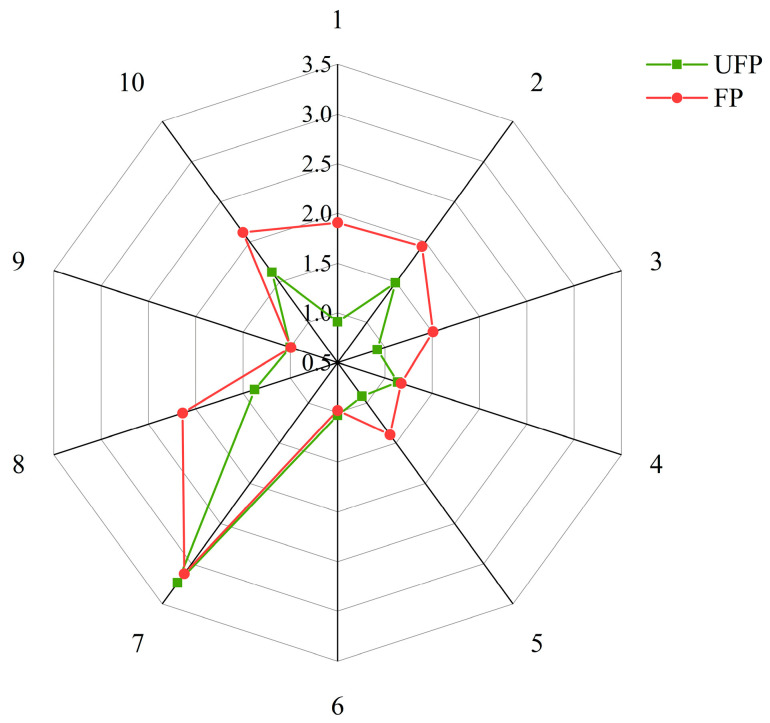
Aroma radar map of pea milk before and after fermentation. UFP: unfermented pea milk; FP: fermented pea milk.

The E-nose responses before and after pea milk fermentation are illustrated in Figure 4. The response values (G/G0) of W1C (aromatic compounds) and W2S (sensitive substances of alcohols, aldehydes, and ketones) increased after the fermentation of pea milk. This showed that fermentation increased the amount of aroma substances in the pea milk and made the flavor substances in the fermented pea milk more prominent. These findings indicate that fermentation effectively enhances the production of volatile flavor substances in pea milk.

### 3.6. Volatile Flavor Components in Fermented Pea Milk

The distinctive flavor of peas is a significant factor that influences their processing into plant-based protein beverages, thus limiting their application in the food industry. In general, the flavor and taste profile of fermented foods are influenced by the generation of new compounds during the fermentation process [35]. For instance, fermented pea milk acquires its distinctive flavor primarily from the presence of esters, acids, alcohols, ketones, and aldehydes that are produced during fermentation [36].

The alterations in aroma compounds within the samples were analyzed using HS-SPME-GC-MS. A total of 64 volatile flavor compounds were detected, containing 15 aldehydes, 15 alcohols, 11 ketones, 5 acids, 8 esters, 2 furans, 3 phenol, and 6 other compounds (Table 3). Regarding the abundance of compounds, aldehydes and alcohols are the most prominent, followed by ketones and esters. In total, 42 common substances were produced in unfermented pea milk, whereas 56 common substances were produced in fermented pea milk, and 23 substances were present only in fermented pea milk. This observation indicates that the fermentation process with Bla80 strain brings about a transformation of substances present in unfermented pea milk, resulting in the production of a variety of new flavor compounds in fermented pea milk. 

After fermentation, the proportion of aldehydes in the total volatile compounds of unfermented pea milk decreased from 75.51% to 47.34% (Table 4). Hexanal, nonanal, octanal, pentanal, and decanal were identified as the primary compounds contributing to the odor, and their concentrations decreased following fermentation (Table 3). On the other hand, heptanal, (E,E)-2,4-decadienal, benzaldehyde, 2-pentenal, and (E,E)-2,4-nonadienal were exclusively detected in the unfermented pea milk. Note in particular, (E,E)-2,4-nonadienal imparted grassy, mushroom-like, and fatty aromas, while (E,E)-2,4-decadienal exhibited fatty aromas profile and has been associated with the characteristic beany aromas [37]. Yi et al. [11] showed that fermentation of *L. plantarum* could reduce hexanal in soy milk, which is consistent with our findings. Aldehydes are reduced or oxidized in the fermentation process to produce acids or alcohols, so the aldehydes tend to decrease after fermentation. LAB can convert caproic aldehyde into caproic acid by fermentation, reduce the taste of beans and grass, and improve the bad taste of beans in pea milk [38].

The fermentation process involving the Bla80 strain yields a substantial quantity of alcohols, which are the end products of amino acid catabolism. Eight alcohols were detected in unfermented pea milk, whereas 14 alcohols were detected after fermentation (Table 3). 1-Hexanol, 1-nonanol, and 1-octanol exhibited the most prominent presence within the unfermented pea milk and were primarily responsible for the undesirable taste of the peas. The fermentation of Bla80 resulted in a decrease in the content of 1-nonanol and 1-hexanol and an increase in the content of 1-octanol. The production of 1-octanol and 1-nonanol can occur through the reduction of octanal and nonanal, respectively, facilitated by ethanol dehydrogenase activity [39]. Indeed, 1-hexanol is a distinctive compound within the flavor profile of legumes, characterized by its grassy aroma. Similarly, 1-nonanols impart a pleasant scent reminiscent of rose and orange aromas [40]. These compounds contribute to the flavor profile of pea milk during fermentation [41]. Furthermore, 1-octen-3-ol is derived from the degradation of unsaturated fatty acids and is a prevalent volatile compound in fermented legumes [42].

In the unfermented pea milk, merely two esters were identified, whereas their number expanded to a total of eight subsequent to the fermentation process (Table 3). Certain acids have the potential to undergo esterification, leading to the formation of ester compounds that contribute to the mild and pleasant flavor of fermented foods, imparting a fruity or floral aroma character [43]. The enhancement of fermented pea milk flavor is attributed to the generation of ethyl acetate (sweet, fruity), ethyl pyruvate (floral and fruity aroma), methyl octanoate (orange aroma), ethyl decanoate (fruity, wine), isoamyl caprylate (fruity), and tetrahydrofurfuryl acetate (fruity) post-fermentation. The presence of esters contributes to the complexity of the product and has the capability to mitigate bitter and unpleasant odors that may originate from fatty acids and amines [44]. Esters offer floral and fruity flavors with a low flavor threshold, effectively masking undesirable flavors [45].

Studies have reported the abundance of furans in pea protein, with particular emphasis on 2-pentylfuran, which is recognized as a characteristic flavor compound of peas [46]. The flavor of 2-pentylfuran and 2-ethylfuran is described as “green and fatty” [47]. After fermentation, the content of furan flavoring substances was reduced, which had a positive effect on improving pea milk flavor (Table 4).

In the current study, ketones are suggested to primarily originate from lipid oxidation in plant-based fermentation products [48]. Compared with unfermented pea milk, the content of ketone flavor compounds in fermented pea milk increased. 3-Heptanone (fruity), (E,E)-3,5-Octadien-2-one (sweet), 1-Penten-3-one (loamy, mushroom), 2,3-hexanedione (sweet), and 3-Penten-2-one (earthy, mushroom aroma) were identified in fermented pea milk. Furthermore, unlike unfermented pea milk, certain ketones (6-Methylhept-5-en-2-one, Geranyl acetone) exhibited higher concentrations in fermented pea milk. 

Moreover, the fermentation process involving Bla80 leads to an increase in the acid content of pea milk. This fermentation generates organic acids, such as palmitic acid, acetic acid, and caproic acid, contributing to the emergence of fresh acidity in fermented pea milk (Table 3).

## 4. Conclusions

This study investigated the viability of Bla80 fermented pea milk to enrich the variety of plant-based beverages. The pH and titratable acidity of fermented pea milk changed with the extension of fermentation time. The metabolites produced during the fermentation of probiotics improved the physicochemical properties, particle size distribution, and rheological properties of pea milk. Probiotic fermentation can effectively reduce the particle size of pea milk, improve the consistency of pea milk, and improve the taste of fermented pea milk. Volatile flavor substances revealed that the mild fermentation of Bla80 decreased the contents of aldehydes and furans, increased the contents of ketones, alcohols, esters, and acids, and improved the flavor quality of pea milk. The results indicated that Bla80 fermentation enhances the functional properties and physicochemical characteristics of pea milk, thereby accelerating its acceptance among consumers and promoting its development as an alternative to animal-based dairy products.

## Figures and Tables

**Table 1:** Electronic nose (PEN 3), sensors name, and their main applications.

Number in Array	Sensor Name	General Description
1	W1C	Aromatic compounds, benzols
2	W5S	Sensitive to nitrogen oxides
3	W3C	Sensitive aromatic components, ammonia
4	W6S	Selective to hydrides
5	W5C	Short-chain alkanes and aromatic components
6	W1S	Sensitive to methyl groups
7	W1W	Sensitive to sulfide
8	W2S	Sensitive to alcohols, aldehydes, and ketones
9	W2W	Sensitive to organic sulfides
10	W3S	Sensitive to long-chain alkanes

**Table 2:** Texture characteristics of pea milk before and after fermentation.

Sample	Hardness (g)	Consistency (g.sec)	Cohesiveness (g)	Work of Cohesion (g.sec)
FP	24.58 ± 0.98 ^a^	244.74 ± 13.21 ^a^	−17.18 ± 0.71 ^a^	−18.16 ± 0.31 ^a^
UFP	21.05 ± 1.91 ^b^	198.76 ± 18.38 ^b^	−15.08 ± 1.89 ^a^	−17.46 ± 1.37 ^a^

UFP: unfermented pea milk; FP: fermented pea milk. The results represented as means ± SD (*n* = 3). Different superscript letters in the table denote significant differences between samples (*p* < 0.05).

**Table 3:** Analysis of flavor substances in pea milk before and after fermentation.

No.	Compounds Name by Classes ^a^	RT/min	Relative Quantities (μg/Kg) ^b^	
			UFP	FP
	Aldehydes			
1	Pentanal	2.58	19.87	1.56
2	Hexanal	4.27	236.11	96.13
3	Heptaldehyde	6.49	4.98	ND
4	2-Hexenal	7.48	3.17	0.86
5	Octanal	9.16	6.16	0.63
6	(E)-Hept-2-enal	10.23	5.52	11.78
7	2-pentenal	10.87	1.51	ND
8	Nonanal	12.01	25.25	13.27
9	(E)-2-octenal	12.89	2.82	7.98
10	(E,E)-2,4-decadienal	21.09	0.33	ND
11	Dodecanal	13.55	0.22	0.90
12	Decanal	14.77	9.57	0.88
13	(E,E)-2,4-nonadienal	18.79	0.35	ND
14	(E)-2-nonanal	15.67	ND	2.84
15	Benzaldehyde	15.37	2.57	ND
	Alcohols			
16	Alcohol	2.21	ND	1.15
17	cis-3-Hexen-1-ol	12.32	2.82	ND
18	1-pentanol	8.98	ND	3.30
19	Trans-2-Octen-1-ol	17.87	ND	1.45
20	1-Octen-3-ol	14.90	3.19	6.74
21	3-Methyl-1-butanol	7.99	ND	0.37
22	2-ethyl-1-hexanol	14.86	1.98	7.51
23	1-nonanol	18.86	5.17	1.87
24	1-hexanol	11.47	18.06	6.34
25	1-octanol	16.50	ND	4.08
26	Benzyl alcohol	17.79	0.30	0.28
27	1-heptanol	14.01	2.80	1.60
28	Linalool	16.30	1.17	2.81
29	1-decanol	21.18	ND	1.29
30	1-Dodecanol	25.47	ND	0.29
	Ketones			
31	2,3-Pentanedione	3.95	1.50	1.14
32	3-Heptanone	5.78	ND	0.50
33	2-Undecanone	17.27	0.29	0.27
34	2-Heptanone	9.05	0.56	ND
35	1-Penten-3-one	9.62	ND	1.92
36	2,3-hexanedione	10.38	ND	3.38
37	6-Methylhept-5-en-2-one	10.69	1.84	4.37
38	3-Penten-2-one	12.49	ND	4.44
39	(E,E)-3,5-Octadien-2-one	15.42	ND	16.09
40	Acetophenone	18.39	0.60	ND
41	Geranyl acetone	23.03	0.51	3.14
	Acids			
42	Acetic acid	14.27	2.33	9.22
43	Caproic acid	23.14	0.39	1.13
44	Nonanoic acid	29.40	ND	0.39
45	Benzoic acid	34.34	ND	0.14
46	Palmitic acid	40.45	0.43	0.52
	Esters			
47	Ethyl acetate	1.75	ND	4.20
48	Ethyl pyruvate	1.82	ND	6.80
49	Methyl octanoate	11.75	ND	0.18
50	Ethyl octanoate	12.97	ND	14.37
51	Ethyl decanoate	18.14	ND	4.36
52	3-methylbutyl octanoate	18.60	ND	0.27
53	Tetrahydrofurfuryl acetate	23.48	ND	4.37
54	Dioctyl phthalate	43.19	0.17	0.64
	Furans			
55	2-Ethylfuran	2.27	2.56	1.55
56	2-pentylfuran	7.35	5.90	2.53
	Phenol			
57	Phenol	26.27	0.18	0.58
58	Eugenol	29.20	3.53	10.69
59	Isoeugenol	32.54	0.28	0.72
	Others			
60	Styrene	8.23	ND	1.91
61	trans-Caryophyllene	12.20	1.73	0.84
62	Iso-caryophyllene	16.70	39.35	9.06
63	Octadecyl vinyl ether	17.38	ND	0.90
64	Alpha-caryophyllene	18.29	5.65	2.49

ND, not detected.; UFP: unfermented pea milk; FP: fermented pea milk. ^a^ Detected aroma compounds. ^b^ Calculated using the internal standard 2-methyl-3-heptanone.

**Table 4:** Quantitative comparison of different chemical classes of pea milk before and after fermentation.

No.	Flavor Volatiles	Relative Quantities (μg/Kg) *		Percentage	
		UFP	FP	UFP (%)	FP (%)
1	Aldehydes	318.43	136.83	74.70	47.34
2	Alcohols	35.49	39.08	8.33	13.52
3	Ketones	5.30	35.25	1.24	12.20
4	Acids	3.15	11.40	0.74	3.94
5	Esters	4.75	35.19	1.11	12.18
6	Furans	8.46	4.08	1.98	1.41
7	Phenol	3.99	11.99	0.94	4.15
8	Others	46.73	15.20	10.96	5.26

* Relative quantities were calculated using the internal standard 2-methyl-3-heptanone. UFP: unfermented pea milk; FP: fermented pea milk.

## Data Availability

The original contributions presented in the study are included in the article, further inquiries can be directed to the corresponding author.
